# *Fibroblast Growth Factor 21* Suppresses Adipogenesis in Pig Intramuscular Fat Cells

**DOI:** 10.3390/ijms17010011

**Published:** 2015-12-23

**Authors:** Yongliang Wang, Xinyi Liu, Liming Hou, Wangjun Wu, Shuhong Zhao, Yuanzhu Xiong

**Affiliations:** 1 Key Laboratory of Agriculture Animal Genetics, Breeding and Reproduction, College of Animal Science, Huazhong Agricultural University, Wuhan 430070, China; ylwang@webmail.hzau.edu.cn (Y.W.); XinyiLiu9@gmail.com (X.L.); mnhouliming@gmail.com (L.H.); 2 Department of Animal Genetics, Breeding and Reproduction, College of Animal Science and Technology, Nanjing Agricultural University, Nanjing 210095, China; wuwangjun2012@njau.edu.cn

**Keywords:** pig, *FGF21*, adipogenesis, IMT, *CEBPB*

## Abstract

*Fibroblast growth factor 21* (*FGF21*) plays an important role in the treatment of disease associated with muscle insulin resistance which is characterized by various factors, such as intramuscular triglyceride (IMT) content. Studies have also shown that *FGF21* inhibits triglyceride synthesis *in vivo*. However, the precise mechanism whereby *FGF21* regulates triglyceride metabolism in intramuscular fat (IMF), which may influence the muscle insulin sensitivity, is not clearly understood. In order to understand the role of *FGF21* in IMF deposition, we performed *FGF21* overexpression in IMF cells by stable transfection. Our results showed that *FGF21* inhibited the key adipogenesis gene mRNA expression of *peroxisome proliferator-activated receptor gamma* (*PPARG*), *CCAAT/enhancer-binding protein* (*CEBP*) family by reducing *lysine-specific demethylase 1* (*LSD1*) expression which led to significant decline in lipid accumulation, and the result was confirmed by Western blot. Moreover, triggered by *FGF21*, parts of the adipokines—*fatty acid-binding protein 4* (*FABP4*), *glucose transporter 4* (*GLUT4*), *adiponectin* (*ADIPOQ*), and *perilipin* (*PLIN1*)—were also down-regulated. Furthermore, *FGF21* gene expression was suppressed by transcription factor *CEBP beta* (*CEBPB*) which contributed strongly to triglyceride synthesis. Taken together, our study is the first to experimentally demonstrate *FGF21* emerging as an efficient blockade of adipogenesis in IMF, thus also providing a new understanding of the mechanism whereby *FGF21* improves insulin sensitivity.

## 1. Introduction

Intramuscular triglyceride (IMT), as an indispensable energy source for skeletal muscles, has been considered to be a robust indicator of muscle insulin sensitivity, which is an essential predictive factor for type 2 diabetes. Larson Meyer *et al.* [1,2,3,4] found that lipid accretion in skeletal muscles contributes to insulin resistance. In addition, ectopic fat accumulation in skeletal muscles is associated with the early pathogenesis of insulin resistance. This has inspired heightened efforts to be made to better understand the precise lipid metabolism in muscles.

Many molecules and pathways are involved in lipid metabolism. *Wnt* signal plays an important role in the regulation of adipocyte differentiation, which was first reported in the MacDougald laboratory [5]. By blocking induction of *PPARG* and *CEBP alpha* (*CEBPα*), *Wnt* signal shows ability in inhibiting adipogenesis. However, disruption of *Wnt* signal leads to spontaneous adipocyte differentiation [6,7,8]. The *Wnt* coreceptor *LRP6* plays a very critical role in the reduction of body mass by reducing nuclear localization of β*-catenin* and inactivation of *forkhead box O1* (*FOXO1*) which promotes adipocyte differentiation [9], resulting in down-regulation of genes involved in adipogenesis [10].

*Kruppel-like factor* (*KLF*) *family* encodes both transcriptional activator and repressor proteins which carry out important roles on differentiation of cells in mammals [11].

It has been known for years that histone deacetylases (*HDACs*) regulate a variety of processes, including growth arrest, differentiation, cytotoxicity, induction of apoptosis [12], and adipogenic transcription factor activity [13]. *HDAC3* controls the circadian rhythm of hepatic lipogenesis. Mice with liver-specific depletion of *HDAC3* reroute metabolic precursors towards lipid synthesis and storage within lipid droplets [14].

In the last decade, *FGF21* as a novel metabolic regulator attracted much attention of scholars. Studies showed that *FGF21* could enhance adipogenesis or attenuate lipolysis [15,16,17,18,19,20]. However, some conflicting data has also been reported. Coskun *et al.* [21] showed that *FGF21* could correct obesity in mice via ameliorating insulin and leptin resistance. Furthermore, Chau demonstrated that *FGF21* was a potential function as a therapy for obesity by activating *AMPK*-*sirtuin 1* (*SIRT1*)-*PGC-1α* pathway [22]. In addition, *FGF21* was reported to suppress the adipogenesis-related genes in liver, *FGF21* had a benefit to fatty liver disease [23], and the treatment of recombinant FGF21 in 3T3-L1 could increase lipolysis [15]. What is more, Hotta [21] demonstrated *FGF21* had opposite roles in different conditions.

Given the situation mentioned above, the functions of *FGF21* in pharmacology and physiology are somewhat discordant. To understand whether and how *FGF21* influences lipid metabolism in intramuscular fat cells, *FGF21* gain-of-function by stable transfection in intramuscular preadipocyte was performed in this study. Our results showed *FGF21* down-regulated the expression of *LSD1* and resulted in the decrease of the adipogenesis-related key genes expression, which resulted in decline of the accumulation of triglyceride in muscles. It has contributed to our understanding of the mechanisms regulating triglyceride metabolism in IMF. Moreover, it also provides another perspective to interpret the pharmacological properties of *FGF21* in the treatment of insulin resistant associated disease, such as type 2 diabetes.

## 2. Result

### 2.1. Low Expression of Fibroblast Growth Factor 21 (FGF21) in Fatty Tissue

**Figure 1:**
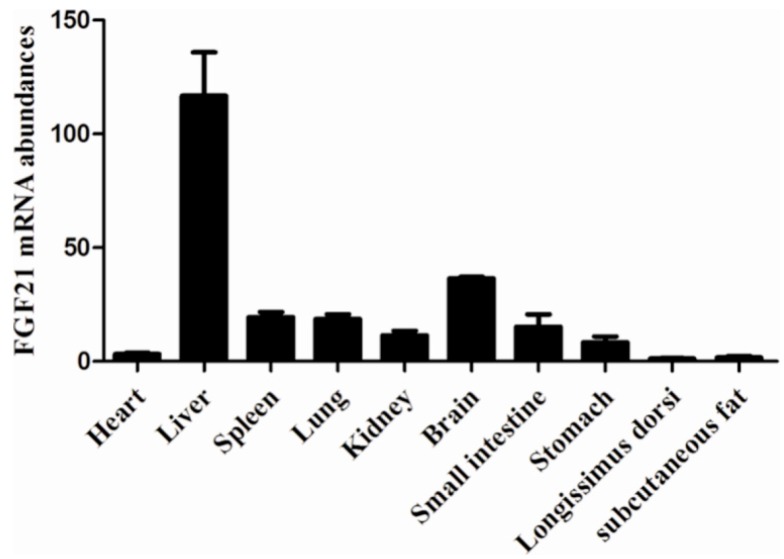
Expression profiles analysis of *FGF21*. The relative expression of *FGF21* in heart, liver, spleen, lung, kidney, brain, small intestine, stomach, longissimus dorsi and subcutaneous fat in three-month Large White pigs and the mRNA level was normalized with *glyceraldehydes phosphate dehydrogenase* (*GAPDH*).

In general, the result of *FGF21* expression profile analysis showed us that the higher expression of FGF21 was found in non-fatty tissues in spleen and brain, especially in liver, rather than in subcutaneous fat. Surprisingly, weaker expression in longissimus dorsi was observed (Figure 1).

### 2.2. Establishment and Identification of the Intramuscular Preadipocyte Cell Line

**Figure 2:**
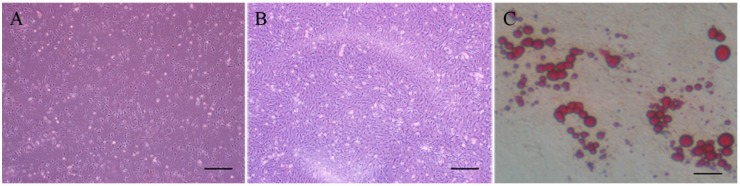
Morphology of the primary cultured cells. (**A**) Morphology of primary cultured pig intramuscular fat cells on the 3rd day (bar = 200 μm); (**B**) Morphology of primary cultured pig intramuscular fat cells on the 8th day (bar = 200 μm); and (**C**) Lipid droplets became jacinth colored by oil red O (bar = 200 μm).

Cells isolated from a three day new born Large White pig were presented as spindle, which were similar to fibroblasts and there were no lipid droplets in the cytoplasm. Oil red staining was used to identify the cells and oil red could be dissolved by fat and oil red presented (jacinth). Oil red staining was performed after the cell differentiation was induced on the 8th day by IBMX + DEX + insulin. A significant amount of lipid droplets generated in the cytoplasm was observed under the inverted microscope which provided direct evidence that the cells we isolated were intramuscular preadipocyte cells (Figure 2).

### 2.3. Cells Transfection and Stable FGF21 Cell Line Acquisition

**Figure 3:**
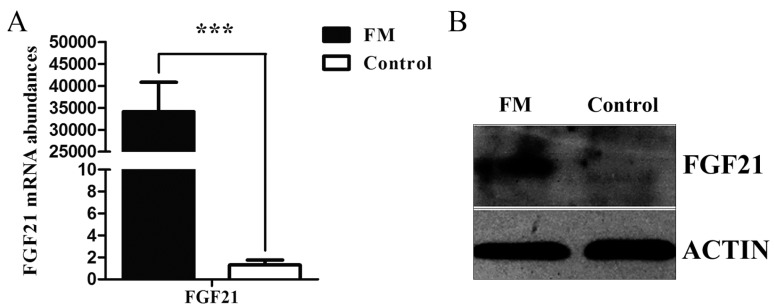
*FGF21* expression in the *FGF21* stable monoclone (FM) cells before cell differentiation. (**A**) qRT-PCR analysis revealed that FM cells had higher *FGF21* mRNA level than that in the control; and (**B**) Western blot analysis showed that the FGF21 protein level was up-regulated significantly in FM cells. Gene expression was normalized to *GAPDH*. Data was presented as mean ± SD, *** for *p* < 0.001.

Because of the low transfection efficiency of pig IMF cells, we performed a stable transfection. After 10 days of screening, we acquired the *FGF21* stable monoclone (FM). The shape of monoclone did not change too much from that of the initial cells. To investigate whether the *FGF21* was up-regulated in FM, qRT-PCR was used to measure the mRNA expression of *FGF21*. Data showed that *FGF21* was weakly expressed in the control, but strongly expressed in FM which was also verified by data of Western blot (Figure 3A,B).

### 2.4. The Function of FGF21 in Adipogenesis

**Figure 4:**
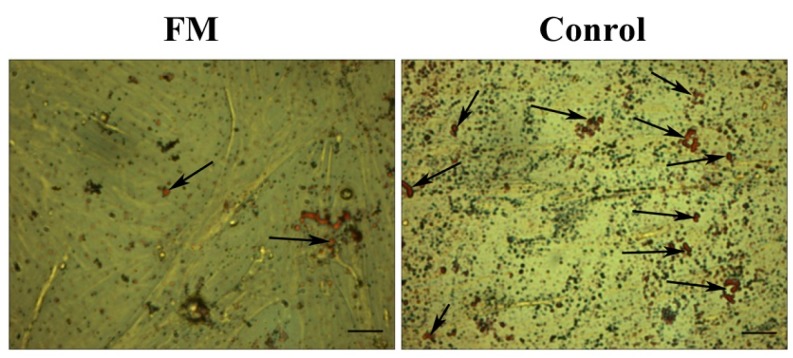
*FGF21* suppressed the accumulation of triglycerides. Oil red staining results showed less lipid droplets in FM cells (**left**) than that in the control (**right**) (bar = 100 μm). The arrows indicated the lipid.

**Figure 5:**
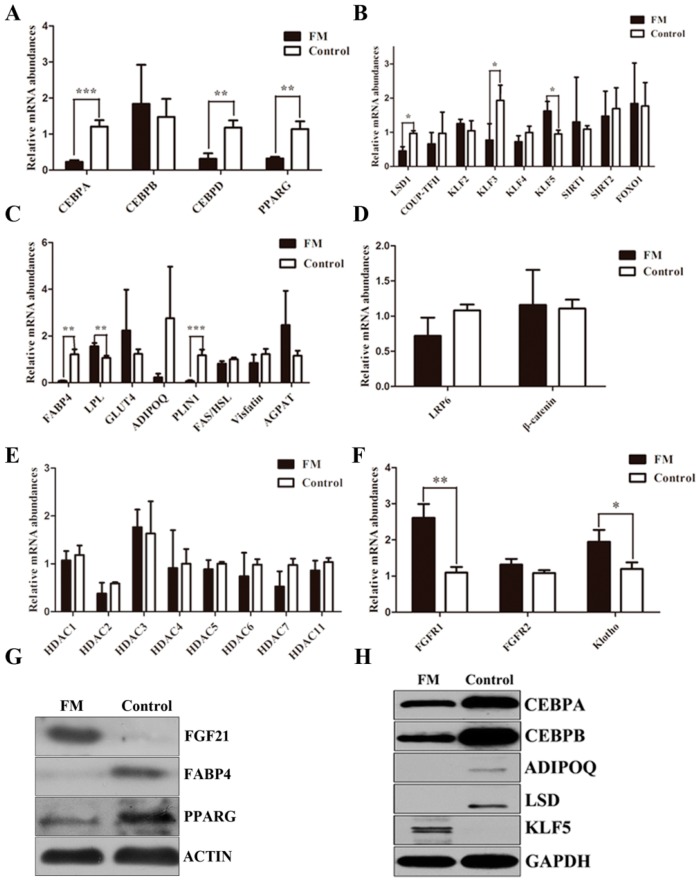
*FGF21* reduced the adipogenesis-related genes expression on the cell differentiation 8th day. The qRT-PCR analysis of *CEBP* family and *PPARG* (**A**); The genes expression regulating *CEBP* family and *PPARG* (**B**); *adipokines* (**C**); *Wnt* signal (**D**); *HDAC* family (**E**) and *FGFRs* signal (**F**) on the cell differentiation 8th day. Gene expression was normalized with *GAPDH*. Data was presented as mean ± SD, * for 0.01 < *p* < 0.05, ** for 0.001 < *p* < 0.01 and *** for *p* < 0.001; Western blot analysis of the adipogenesis-related genes on the cell differentiation 8th day. The gene expression was normalized with ACTIN (**G**) and GAPDH (**H**).

To specifically evaluate a potential role for *FGF21* in adipogenesis, we examined the effect of FGF21 on the 8th day of adipocyte differentiation. We found that the mRNA expression of the key genes *PPARG*, *CEBPA* and *CEBPD* involved in adipogenesis was down-regulated dramatically in FM compared with the control, which was the same with down-regulation of the protein level. Interestingly, no change occurred in the *CEBPB* mRNA level, but the protein of *CEBPB* declined strongly. In short, *FGF21* suppresses adipogenesis as assessed by oil red O staining (Figure 4 and Figure 5).

To further explore the mechanism above, some molecules participating in the regulation of the key adipogenic genes were also analyzed. There were no significant changes in the mRNA levels of *KLF2*, *SIRT1*, *SIRT2* and *FOXO1*, which could regulate *PPARG* expression. *Chicken ovalbumin upstream promoter-transcription factor* *II* (*COUP-TF* *II*), also known as *NR2F2*, played an important role in the function of *CEBPA*. There was no significant difference in the *COUP-TF II* expression between FM and control. The same level was found in mRNA of *KLF4* which participated in *CEBPB* expression. Unexpectedly, there was a higher level in *KLF3* mRNA and a lower level in *KLF5* mRNA.

Compared with *FGF21* expression of FM and control, *FAS* and *hormone-sensitive lipase* (*HSL*) mRNA levels were both up-regulated. However, the ratio of *FAS* and *HSL* was down-regulated although it did not reach a significant level. We also found that *AGPAT2* and *visfatin* mRNA levels did not change.

In the circumstances that *FGF21* suppresses adipogenesis, we would expect *FGF21* could down-regulate the adipocytokines. So in order to find out the role of *FGF21* on adipocytokines, those encoding *FABP4* (also known as *adipocyte protein 2*, *ap2*), *GLUT4*, *ADIPOQ*, and *PLIN1* were evaluated. We proved *FABP4* and *PLIN1* declined dramatically in mRNA level. However, there was no significant change in *GLUT4* and *ADIPOQ*. Western blot analysis demonstrated FABP4 and ADIPOQ protein were rapidly decreased. To identify *FGF21* candidate pathway with the potential to modulate adipogensis, we assessed the regulatory program governing inhibition of adipogenesis by the canonical *Wnt*/β*-catenin* signal. We analyzed the gene expression of *LRP6* and β-*catenin* in mature adipocyte that was induced to differentiate on the 8th day. QRT-PCR analysis demonstrated *FGF21* did not change the mRNA expression of *LRP6* and β*-catenin*. Due to the important role of *HDACs* in adipogensis, we conducted the necessary test to determine whether *FGF21* affects the mRNA expression of *HDACs*. However, all of the family of *HDACs* in our study did not change significantly. Yet, *LSD1*, a crucial regulator in histone methylation of *CEBPA*, decreased in both mRNA and protein levels (Figure 5).

**Figure 6:**
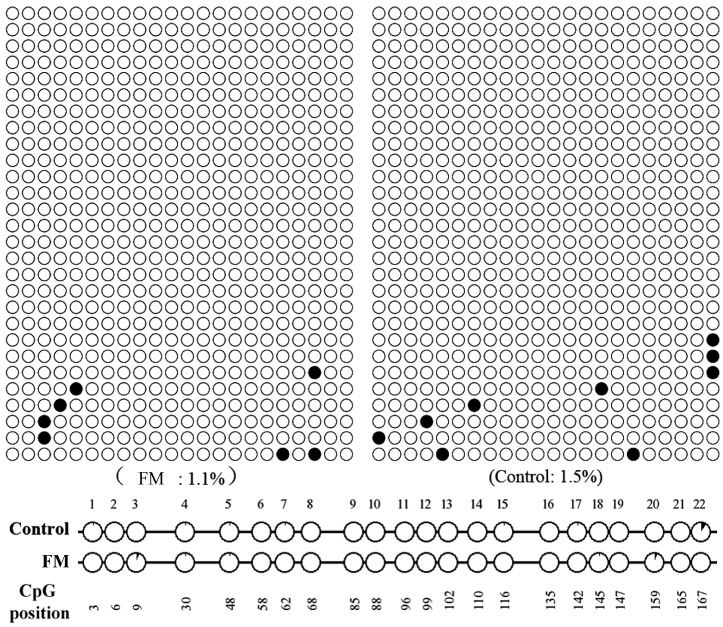
Methylation analysis of *CEBPA* promoter. No significant difference of methylation level between FM and the control was found in the region from −172 to +2 bp in *CEBPA* promoter with the Quantification Tool for Methylation Analysis on line.

Given an important role of the gene promoter methylation for gene expression and to figure out whether *FGF21* influenced the methylation level of *CEBPA* promoter to regulate *CEBPA* and further regulate *PPARG* expression, *CEBPA* promoter methylation level was also detected. The level of methylation of the *CEBPA* promoter did not change with the Quantification Tool for Methylation Analysis on line (Figure 6).

### 2.5. The Inhibitory Effect of the Transcription Factor CEBPB in FGF21 Expression

**Figure 7:**
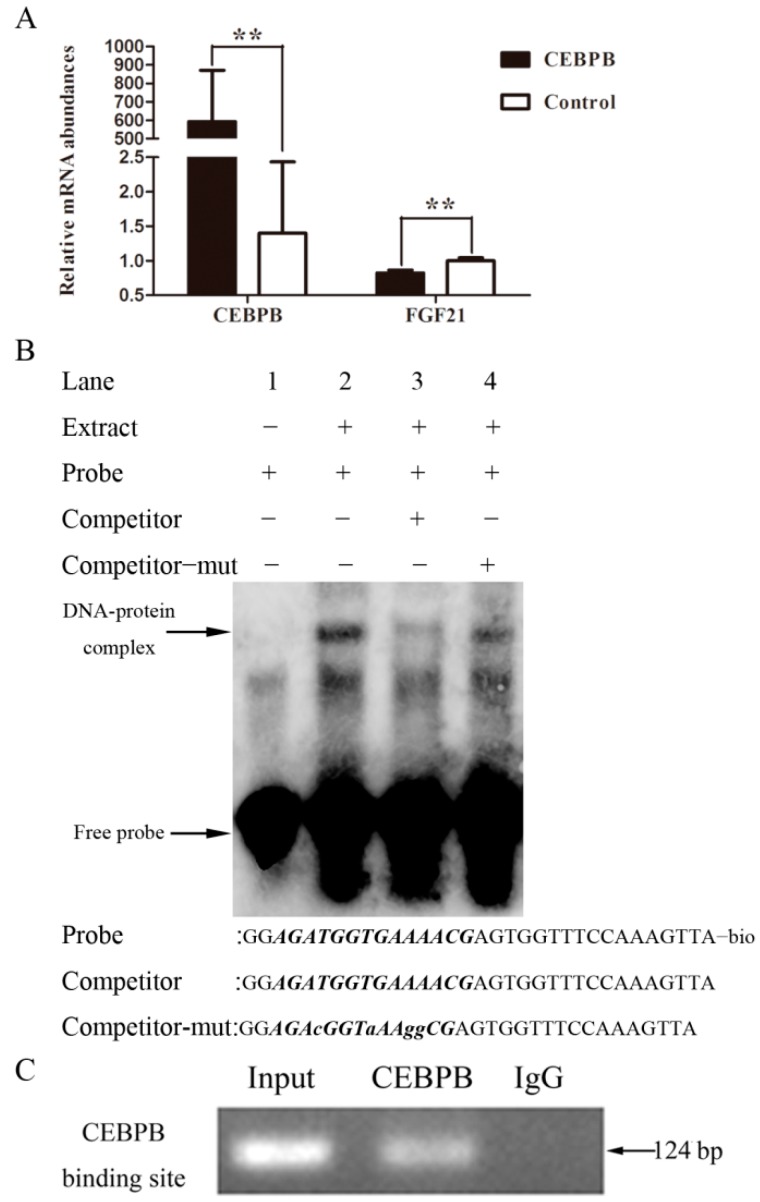
The transcription factor *CEBPB* suppressed *FGF21* expression by binding to *FGF21* promoter. QRT-PCR analysis showed *CEBPB* reduced *FGF21* mRNA level. ** for 0.001 < *p* < 0.01 (**A**); The electrophoretic mobility shift assay (EMSA) was used to simulate the binding of *CEBPB* with *FGF21* promoter *in vitro* (**B**); The chromatin immunoprecipitation (CHIP) assay was performed to detect the binding of *CEBPB* with *FGF21* promoter *in vivo* (**C**), total chromatin and normal mouse IgG were used as the input and the negative control respectively.

The porcine *CEBPB* cDNA fragment was inserted into plasmid pcDNA 3.1(+), named pcDNA3.1-*CEBPB*, which was transfected into PK15 cells using Lipofectamine 2000 (Invitrogen, New York, NY, USA), and then the total RNA was extracted and reversed. The result of qRT-PCR suggested CEBPB suppressed FGF21 expression in the mRNA level (Figure 7A).

To investigate whether the transcription factor *CEBPB* can bind to the promoter of *FGF21* to regulate the gene expression, the electrophoretic mobility shift assay was carried out. Our results showed that a DNA-protein complex emerged when the nuclear extracts of IMF cells were incubated with *CEBPB* probe (lane 2). However the complex was weakened sharply in the presence of 50× cold probe (lane 3) compared with that in 50× mutation cold probe (Figure 7B).

CHIP analysis was preformed to verify whether *CEBPB* binds to the promoter of *FGF21*. Our results demonstrated one single DNA band was obtained on agarose gel electrophoresis from PCR product when PK15 cells were incubated with anti-CEBPB, but no band appeared with Normal Mouse IgG (Figure 7C).

## 3. Discussion

Given the essential role of IMT content in insulin resistant associated disease, the complicated and orderly expression of genes in muscles had been shown to be involved in triglyceride metabolism and their underlying action mechanism in muscles was beginning to be elucidated. Our new finding that FGF21 suppressed adipogenesis in muscles, however, provides a new insight for the treatment of insulin resistant associated disease.

*FGF21* was known as a metabolic regulator taking part in glucose metabolism and lipid metabolism and had the ability to treat insulin resistant associated disease [23,24,25,26,27]. However, little was known about its effect on triglyceride metabolism in intramuscular triglycerides, which emerged as a hallmark of insulin resistant associated disease [28,29,30]. In order to understand the effect on IMF, we performed *FGF21* overexpression in IMF cells and evaluated the expression of the relative genes participating in triglyceride metabolism.

*PPARG* was a key gene in adipocyte differentiation, activated most genes involved in adipogenesis directly [31] and played a critical role in fat deposition in mammals [32]. Considering the key role of this molecule, we first evaluated the expression of *PPARG*. The sharp decline of *PPARG* was observed, demonstrating *FGF21* suppressed adipogenesis by decreasing the expression of *PPARG* directly or indirectly. As is well known, many factors influencing adipogenesis ultimately also affected the activity of *PPARG* [13]. *CEBPB* and *CEBPD*, induced by adipogenic induction, targeted the promoters of the genes encoding PPARG and CEBPA directly. Then, the interactive activation between *PPARG* and *CEBPA* induced a range of genes expressed in mature adipocytes [13]. In order to figure out how *FGF21* decreased *PPARG* expression whether or not through down regulating the expression of *CEBP* family, the mRNA levels of these genes were measured. The result suggested the *CEBPB* and *CEBPD* protein levels were decreased significantly by the action of *FGF21*, although the mRNA of *CEBPB* did not change significantly, which together suggested that the decline of the *CEBP* family decreased the expression of *PPARG* expression.

Unexpectedly, the interaction between *FGF21* and *CEBPB* was mutual. *FGF21* down-regulated the expression of the transcription factor *CEBPB* as mentioned above. *CEBPB* led to the reduction of *FGF21* expression by binding to the promoter of *FGF21* based on the EMSA and CHIP analysis, thus demonstrating the biological function of *FGF21* was constrained by *CEBPB.* This also explained the phenomenon of the low level of *FGF21*, but the high level of *CEBPB* in fatty tissue.

To figure out the deep interaction relation of *FGF21* and *CEBP* family which contributed to the declined expression of *PPARG*, we also measured the expression of the genes involved in the key genes expression in triglyceride metabolism mentioned above. *SIRT1*, acting as a *PPARG* co-repressor, impaired adipogenesis [33]. *SIRT2* reduced *FOXO1* acetylation and phosphorylation, led to an increase in the nuclear localization of *FOXO1*, and resulted in inhibiting *PPARG* indirectly [34]. We demonstrated *FGF21* indulged in the action of sirtuin during adipocyte differentiation. *COUP-TF* *II*, activated by *Wnt/*β*-catenin*, recruited the *SMRT* corepressor complex to *PPARG* promoter to repress *PPARG* gene expression [35]. We showed that there were no obvious changes observed in expression of *LRP6*, β*-catenin* and *COUP-TF II*, which suggested *FGF21* appeared to have no effect on the *Wnt/*β*-catenin*-*COUP-TF ii*-*PPARG* pathway.

*KLF2* inhibited *PPARG* and *CEBPA* expression in the process of 3T3-L1 cell differentiation [36]. By binding to *CEBPA* promoter *in vivo*, *KLF3* repressed the transcription of *CEBPA* [37]. *KLF4*, together with *Krox20*, bound directly to the *CEBPB* promoter and transactivated *CEBPB* reporter cooperatively [38]. *KLF5* regulated adipocyte differentiation by activating *PPARG* promoter [39]. Our data showed there were no significant changes in *KLF2* and *KLF4*. The decrease of *PPARG* and *CEBPA* was not altered although an increase in *KLF5* and decrease in *KLF3* were observed.It was possible that the sharp decline of *CEBPB* and *CEBPD* attenuated the activity of *KLF3* and *KLF5*.

It is worth noting that *LSD1* expression was dramatically reduced compared with the control The result, together with the reduction of *CEBPA* in both mRNA and protein, was consistent with a previous finding that *LSD1* is necessary for adipogenesis by regulating adipogenic transcription factor *CEBPA* [40]. It was possible that *FGF21* realized its biological function in triglyceride metabolism by reducing LSD1.

In addition, ADIPOQ was believed to be produced exclusively by mature adipocytes and induced by *PPARG* and *CEBPA* [13,41]. *FABP4* was also a major player involved in fatty acid metabolism and appeared to be necessary for the differentiation of IMF accretion in pigs [42]. *PLIN1* was established for a distinct role in regulating both TAG storage and lipolysis in adipocytes and was regarded as a candidate gene contributing to human obesity [43]. In our research, *FABP4* and *PLIN1*, except *GLUT4*, were down-regulated in FM significantly which was likely to be caused by the reduction of *PPARG* and *CEBPA*.

*FAS* played an important role in fat deposition, which could catalyze acetyl CoA and malonyl CoA synthesis of fatty acid [44,45]. *HSL* is a limiting velocity enzyme in fat hydrolysis, playing an opposite role to *FAS* [46]. Our results demonstrated that *HSL* mRNA level was up-regulated and *Visfatin* mRNA level was down-regulated, which indicated *FGF21* promotes lipolysis by strengthening the fat hydrolysis process. Several studies have reported that the ratio of *FAS* and *HSL* was positively correlated with intramuscular fat content [47]. In our study, the ratio of *FAS* and *HSL* was down-regulated in FM, but did not reach a significant level. *AGPAT2* converted lysophosphatidic acid to phosphatidic acid, a crucial step in synthesis of triglycerides [48]. However, the level of *AGPAT2* mRNA did not change as obtained from our data.

In order to better understand the mechanism of *FGF21* affected adipogenesis in IMF cells, we analyzed relative gene expression in *FGF21* signal. *FGF21* did not bind *FGFRs* directly. So an interaction partner is required for *FGF21* to perform its function [25]. *Klotho* was a crucial cofactor in *FGF21* signals. Firstly, *FGF21* binds to *Klotho* via its C-terminus. Next *FGF21* contacts *FGFRs* through its N-terminal [49]. In our study, *FGFR1* and *Klotho* were significantly up-regulated, and the mRNA level of *FGFR2* was also increased to some extent. Our results suggested that *FGF21* may be a critical factor affecting IMF by inhibiting gene expression involved in lipogenesis and activating *FGF21* signal, which well matched the result of *FGF21* expression profiles analysis and explained the low expression level of FGF21 in fatty tissues and the high expression level in non-fatty tissues. As for the low expression in muscle tissues, such as longissimus dorsi, the method of modern breeding towards a high content of intramuscular fat may lead to the occurrence of this phenomenon. In addition, our results were consistent with the report by Alexei *et al.* [25], which further confirmed the role of *FGF21* in adipogenesis.

**Figure 8:**
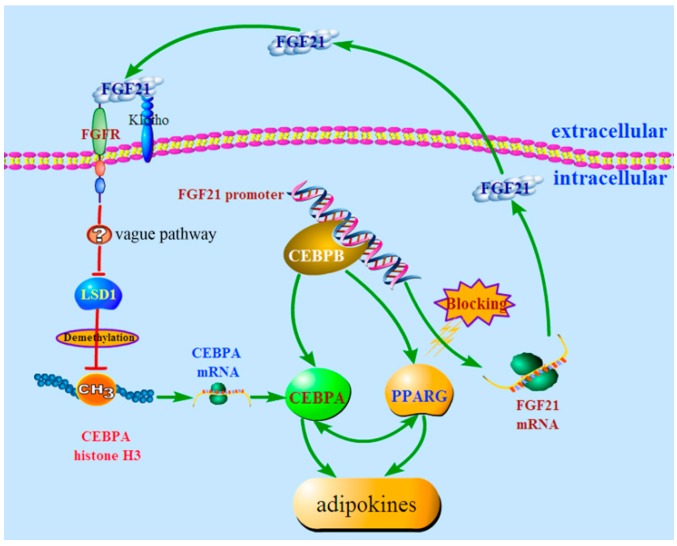
The proposed mechanism of *FGF21* action in triglyceride metabolism. Briefly, *FGF21* suppressed adipogenesis by reducing *LSD1* expression, and in the meantime, *FGF21* expression was repressed by the transcription factor *CEBPB*. The red line represented for the action of inhibition and the green line represented for the action of activation.

Herein, we believe our study to be the first to elaborate on the clear biological function of FGF21 on triglyceride metabolism in IMF. In brief, triggered by *FGF21*, the *FGF21* signal was activated, and then *LSD1* was attenuated, which led to the reduction of *PPARG* and *CEBPA* without changing the methylation level of *CEBPA* and resulted in down-regulation of adipokines and the accumulation of triglycerides in IMF (Figure 8). Our results offer new understanding on FGF21 as a candidate in the treatment of insulin resistant associated diseases.

## 4. Experimental Section

### 4.1. Ethics Statement

All animal experiments were carried out according to Huazhong Agricultural University Animal Care and Use Committee Guidelines.

### 4.2. Expression Profile Analysis of FGF21

The total mRNA from heart, liver, spleen, lung, kidney, brain, small intestine, stomach, Longissimus dorsi, and subcutaneous fat of three-month Large White pigs was extracted by trizol (Invitrogen, New York, NY, USA) and reversely transcribed by Prime Script RT Reagent Kit with gDNA Eraser (TAKARA, Dalian, China). The qRT-PCR was used to analyze FGF21 mRNA expression levels.

### 4.3. Isolation and Identification of Intramuscular Preadipocyte

A three-day new born Large White pig was washed with 0.3% benzalkonium bromide and then the tissue longissimus dorsi (LD) was taken out. The tissue was digested by collagenase type II for 2 h, then passed through a 400 screen mesh filter and centrifuged 5 min at 1500 r/min. Next the precipitate was resuspended by DMEM/F12 with 15% fetal bovine serum (complete medium), and then the medium was changed after 2 h. After confluence, the medium was changed to the complete medium with 0.5 mmol/L 3-isobutyl-1-methyl xanthine (IBMX), 1 μmol/L dexamethasone (DEX), 10 mg/L insulin for 48 h, and then was changed to the complete medium with 10 mg/L insulin for 48 h. Finally, the complete medium was changed with no drug mentioned above for 8 days.

When the preadipocytes were induced into mature adipocyte, the complete media was discarded and the preadipocytes were washed 3 times with phosphate buffered saline (PBS). Subsequently, the cells were fixed by 10% paraformal dehyde for 1h at room temperature, then moderate oil red O was added for 1h at room temperature after washing 3 times with PBS, finally the cells were washed with PBS and observed under an inverted microscope Observer.A1 (ZEISS, Oberkochen, Germany).

### 4.4. Stable Transfection

A 627bp cDNA fragment of *FGF21* from pigs was cloned and inserted into plasmid pcDNA 3.1(+), named pcDNA3.1-*FGF21*. Lipofectamine 2000 (Invitrogen, New York, NY, USA) was used to perform cell transfection and G418 was also used in order to get a stable cell line. Firstly, a set of gradients (100, 200, 300, 400, 500 and 600 ng/mL) of G418 were set up to determine the optimal concentration (300 ng/mL). Cell transfection was performed when cells extended to the third generation. Cells were digested by 0.25% trypsin after 6 h, and then inoculated into 96-well plates and only one cell was put into one well. After the cells were adherent, a certain amount of G418 was added into the media to 300 ng/mL. The screening medium was changed every two days until the monoclone appeared.

### 4.5. RNA Extraction and qRT-PCR

Total RNA was extracted using DNA/RNA/protein isolation kit (OMEGA, Norcross, GA, USA) according to the protocol of the manufacturer. The integrity of the total RNA was detected by 1.2% agarose gel electrophoresis and the concentration was determined using NanoDrop 2000 (Thermo, New York, NY, USA). Then Prime Script RT Reagent Kit with gDNA Eraser (TAKARA, Dalian, China) was used to perform RT-PCR and next CFX96 Real-Time System (Bio-Rad, Berkeley, CA, USA) was used to perform qRT-PCR. The primers for qRT-PCR were designed by Primer Premier 5, which are listed in Table 1.

**Table 1:** Primer Sequences for qRT-PCR.

Genes	Primer Sequences (5′–3′)	Length of Amplicon (bp)
*FGF21*	F: ACTGTGGGTCCCTGTGCTG R: ATCCGTGTAGAGGTATCGTTGG	118
*PPARG*	F: TCCCGCTGACCAAAGCAAAGGC R: CCACGGAGCGAAACTGACACCC	195
*FABP4*	F: GAAAGTCAAGAGCACGATAACC R: CAAGATACATTCCACCACCAAC	124
*FAS*	F: TTACACCTCCCTCAACTTCCG R: GGCACCATTCCCATCACG	153
*HSL*	F: GTCTTTGCGGGTATTCGG R: GAGTTCGGCCAGGTTGTG	228
*Visfatin*	F: TGCCTTTGGTTCTGGTGG R: GCGTAATGAGGTGCTGCTTC	354
*LPL*	F: TAACGAACCCGACCAGCATC R: TACACCACCGCCACAGCAA	140
*FGFR1*	F: GAATCGGAGGCTACAAGGTC R: GATAGAGTTACCCGCCAAGC	305
*FGFR2*	F: CTGCCGCCAACTCTGTCA R: CGGATGGAACCACGCTTT	165
*Klotho*	F: TCATCCTGTCACCGTTTATTC R: CCTCCACCTGAAATGCTCC	169
*CEBPA*	F: CGGTGCGTCTAAGATGAGG R: AGCGGTGAGTTTGCGTTT	120
*CEBPB*	F: AGCCTGTCCACATCCTCG R: CACGGTCTTCTTGGTCTTACTC	135
*CEBPD*	F: TCAAACACGCCGAACTACAC R: GAGCAAAGGGAAAGCAAATC	207
*GLUT4*	F: TGTTGCGGATGCTATGGG R: GGGTTTCACCTCCTGCTCTAA	169
*ADIPOQ*	F: CTGGCGAGAAGAGTGAGA R: TGCTGAACGGTAGACATAGGC	158
*AGPAT2*	F: GTTCGTCCGCTCCTACAA R: CAGGATGCTCTGGTGGTTG	112
*PLIN1*	F: GTGCCAGGAACAGCAACAG R: GGGCTCTACCACCTTCTCATC	194
*FOXO1*	F: AAGACCGCTTTACAAGTGCC R: TCTCCATCCATGAGGTCGTT	200
*LSD1*	F: AGCCAGTTGACAGTGAGGAAT R: CTGAAGCGGTGTAGCGAAC	109
*LRP6*	F: GCAGGGTGGAATGAATGTG R: GAGCAGGAAAGTAGTTGGAGC	150
β *-catenin*	F: AAGCAGGTGGATCTATTTCATG R: AGCATTGTATCACAGCAGGTTA	159
*GAPDH*	F: GGGCATGAACCATGAGAAGT R: AAGCAGGGATGATGTTCTGG124	230
*KLF2*	F: GGCGAGAAGCCCTAACACT R: CGCACAGATGGCATTGGA	121
*KLF3*	F: CGCACAGATGGCATTGGA R: CAGCACATTCCTCCCAGTTA	184
*KLF4*	F: CGGAGGGAGACGGAGGAGTT R: TGAAGCCGACGAGGACACG	108
*KLF5*	F: GAAGGGTGCGACTGGAGGTT R: TGTGCTGGGCGAGGTGAT	133
*COUP-TF* *ii*	F: CAAGGCCATAGTCCTGTTCACC R: CGTACTCTTCCAAAGCACACTGG	101

The primers of *SIRT1* and *SIRT2* are referred to in the literature [49], and for the HDAC family, please see our laboratory published date [50], F for forward and R for reverse.

### 4.6. CEBPA Promoter Methylation Analysis

Genomic DNA from the control and FM induced cell differentiation for the 8th day was extracted using DNA/RNA/protein isolation kit (OMEGA), and then treated with sulfate using a Methylation-Gold Kit (ZYMO, Orange, CA, USA) according to the operation manual. The primers used for amplification of bisulfite-treated DNA were designed and the sequences were forward primer: AGAGGAGAGGGTTTTATTAGGGAT and reverse primer: CTCCATAAAAAAACTAAAATCCTCC. The region of *CEBPA* promoter from −172 to +2 bp was amplified for sequencing. The PCR amplification profile was: 94 °C for 4 min followed by 35 cycles of 94 °C for 30 s, 58 °C for 30 s, and 72 °C for 30 s, with a final extension at 72 °C for 5 min. The Quantification Tool for Methylation Analysis on line was used for *CEBPA* promoter methylation analysis.

### 4.7. Western Blot

The protein was extracted from the positive monoclone using DNA/RNA/protein isolation kit (OMEGA). A total of 40 micrograms of protein for each sample was loaded into 12% SDS-polyacrylamide gel lanes. Proteins were transferred to PVDF membranes after electrophoresis, and then the membranes were blocked by 5% skimmed milk (1g skimmed milk in 20 mL TBST). The blocked membranes were incubated with primary antibodies anti-FGF21 (1:1000; Abcam, Cambridge, UK), anti-PPARG (1:1000; Abcam, UK), anti-FABP4 (1:1000; Abcam, UK), anti-KLF5 (1:1000; Abcam, UK), anti-CEBPA (1:1000; Santa Cruz, CA, USA), anti-CEBPB (1:1000; Santa Cruz), anti-KDM1A (1:1000; Abcam, UK), anti-ADIPOQ (1:1000; Santa Cruz), anti-GAPDH (1:1000; Abcam, UK) and anti-beta-actin (ACTIN) (1:1000; Santa Cruz) overnight at 4 °C, then treated with labeled-horseradish peroxidase-conjugated (HRP) secondary antibody. Finally, photographic plate was used to expose and develop films.

### 4.8. Electrophoretic Mobility Shift Assay (EMSA)

Commercially-available Light Shift Chemiluminescent EMSA Kit (Thermo, New York, NY, USA) was used to perform electrophoretic mobility shift assay. Nuclear protein of pig IMF cells was extracted with Nucleoprotein Extraction Kit (Active Motif, Carlsbad, CA, USA). Oligonucleotides (Sangon, Shanghai, China) corresponding to the *CEBPB* binding sites of the *FGF21* promoter were annealed into double strands. All the reagents in every group were added according to the manufacturer’s instructions. After being incubated for 15 min at room temperature, 20 μL of each mixture was loaded onto the 6% polyacrylamide gel until the bromophenol blue dye had migrated approximately 3/4 down the length of the gel. The transfer time was 45 min at 380 mA (~100 V). Finally, the nylon membrane was scanned with Automated BioSpectrum Imaging System (UVP).

### 4.9. Chromatin Immunoprecipitation (CHIP) Assay

CHIP assay was carried out using a commercially available CHIP Assay Kit (Beyotime, Shanghai, China). After incubating 1% formaldehyde at 37 °C for 10 min, PK15 cells were washed and harvested. Then, the nuclear lysates were sonicated at a setting of 20 times for 10 s pulses using the Scientz-IID (Scientz, Ningbo, China). The chromatin was immunoprecipitated with *CEBPB* antibody (Santa) at 4 °C overnight and the control, under the same experimental condition, was immunoprecipitated with Normal Mouse IgG (Millipore, Billerica, MA, USA). Finally, the immunoprecipitated DNA was purified by the AxyPrep PCR Cleanup Kit (Axygen, Hangzhou, China). One pair of oligonucleotide primers was synthesized to amplify the DNA fragments of *CEBPB* binding sites in the *FGF21* promoter.

### 4.10. Statistical Analysis

Student t-test was used for qRT-PCR statistical analysis and all the data were presented as mean ± SD. Fisher’s exact test was used for Methylation Analysis. 0.01 < *p* < 0.05 was defined as significance, 0.001 < *p* < 0.01 was defined as very significant and *p* < 0.001 was defined as of great significance.

## 5. Conclusions

In this study, we isolated intramuscular preadipocytes from pig longissimus dorsi, and constructed stable transfection intramuscular preadipocytes cell line of pig *FGF21*. We evaluated the expression of the genes involved in adipogenesis when the cell induced adipogenic differentiation on the 8th day. Our final analysis conclusions can be summarized as follow:

1. *FGF21* suppressed adipogenesis by reducing *LSD1* expression.
2. The transcription factor *CEBPB* can bind to the promoter of *FGF21* directly, and attenuates the expression of *FGF21*.
